# PROTOCOL: Prevalence and Risk and Protective Factors for Radicalization Among School‐Aged Youth: A Systematic Review

**DOI:** 10.1002/cl2.70041

**Published:** 2025-04-24

**Authors:** Anthony Petrosino, Claire Morgan

**Affiliations:** ^1^ George Mason University Center for Evidence-based Crime Policy Alexandria Virginia USA; ^2^ Independent Researcher Sun Valley Idaho

## Abstract

This is a protocol for a Campbell systematic review. The objectives are to synthesize published and unpublished scientific literature on (1) the prevalence of and (2) the risk and protective factors for school‐aged youth radicalization. In the nascent field of research on radicalized youth, a priority of the review is to examine the breadth of empirical evidence on prevalence and risk and protective factors, and to identify gaps and opportunities for further research. Our research questions are: Part 1: Prevalence: What is the prevalence of radicalization among school‐aged youth? How does the prevalence of radicalization of school‐aged youth vary over time and by type of radical ideology (e.g., religiously motivated, far left and far right, ethno‐nationalist, racially and ethnically motivated, gender‐based, online conspiracy‐based)? Part 2: Risk and Protective Factors What are the cognitive and behavioral risk and protective factors for school‐aged youth radicalization? How do the cognitive and behavioral risk and protective factors for youth radicalization vary by type of radical ideology (e.g., religiously motivated, far left and far right, ethno‐nationalist, racially and ethnically motivated, gender‐based, online conspiracy‐based)?

## 1 Background

### 1.1 The Problem, Condition, or Issue

Youth radicalization has become a significant global concern with implications for security and social cohesion in many countries. Adolescence is a turbulent time that can generate psychological vulnerabilities that can lead to youth being attracted to extremist ideologies and groups (Bronsard et al. 2022). Extremism refers to “the unlawful use or threat of force or violence… in furtherance of political or social agendas” (Federal Bureau of Investigation & Department of Homeland Security 2023, 4). Recent research shows an upward trend in extremist attitudes among adolescents, which can lead to engagement in extremist activities (Harpviken 2020). Not all radicalization is necessarily negative or a precursor to violent extremism (United Nations 2016), such as resistance actions within the context of an oppressive regime. However, violent propaganda has deeply infiltrated social media, and youth are increasingly participating in extremist activities, which may include online interactions with known extremists, traveling to conflict zones, recruiting others, or supporting attacks (Federal Bureau of Investigation 2016). These trends indicate a growing threat within schools and communities, emphasizing the need for proactive measures to address and prevent youth radicalization (Federal Bureau of Investigation 2016).

Preventing and combatting radicalization is a key priority for governments worldwide and for international organizations that include this aim in their anti‐terrorism^1^ frameworks, such as the United Nations Global Counter‐Terrorism Strategy (United Nations 2016), the European Union Counter‐Terrorism Strategy (Council of the European Union 2005), and the U.S. Department of Homeland Security's Strategic Framework for Countering Terrorism and Targeted Violence (U.S. Department of Homeland Security 2017). Programs aimed at preventing and combating attempts to recruit, radicalize, and mobilize individuals to carry out violence on behalf of a group or ideology are essential (Erdemandi et al. 2024).

The U.S. Department of Homeland Security (2017) defines radicalization as “The process through which an individual changes from a non‐violent belief system to a belief system that includes the willingness to actively advocate, facilitate, or use unlawful violence as a method to effect societal or political change” (p. 538). Radicalization is thus a process toward extremism. Some common extremist ideologies and organizations that youth may orient toward, as laid out by Jones and Dawson (2023), include religiously motivated extremism such as Salifi‐Jihadist, ISIS, and other Islamist extremist groups or extreme factions within Christianity, Hinduism, and Buddhism. Far left and far right ideologies that might attract youth include white supremacist, neo‐Nazi, or anti‐governmental militia or sovereign citizen movements on the right and anti‐fascist and anarchist groups such as accelerationists on the left. Ethno‐nationalist and regional separatist movements also involve youth, as well as racially and ethnically motivated violent extremism. In addition, involuntary celibate (Incel) and gender‐based extremism ideologies and online conspiracy‐based groups such as QAnon can contribute to youth radicalization. Each of these streams has unique appeal points for youth, often tapping into feelings of identity, purpose, alienation, or grievance. Social media platforms, online forums, and encrypted apps play a critical role in spreading these ideologies and recruiting young people, often blurring the lines between different extremist ideologies.

McCauley and Moskalenko's (2017) two‐pyramid model (TPM) of cognitive and behavioral radicalization presents a framework for understanding radicalization through two distinct but interrelated pyramids. The cognitive pyramid focuses on the progression of beliefs and attitudes toward radical ideologies—from neutral attitudes, through sympathetic and supportive ones, to a commitment to radical beliefs. The behavioral pyramid illustrates the progression from legal and nonviolent support and activism, through illegal but nonviolent actions, to violent extremist acts. The two pyramids are connected but not necessarily sequential; thus, an individual can hold radical beliefs without engaging in radical actions, and vice versa.

Another frame through which to understand youth radicalization involves the distinction between radicalization and mobilization to violence. Radicalization and mobilization can be seen as distinct but often intertwined processes (Canadian Security Intelligence Service n.d.), with radicalization being the process of change from a non‐violent system of beliefs to one that views violence as a legitimate means to advance goals, and mobilization being characterized by shifts in behavior indicating preparatory steps to engage in an act of violence (U.S. Department of Homeland Security 2017). In addition, engagement and re‐engagement are often referenced in the radicalization literature as initial phases in which an individual becomes involved with an extremist group or adopts extremist beliefs before radicalization and mobilization (Horgan 2008). Engagement can be influenced by social networks, external influences, personal grievances, and ideological commitment, among other factors.

A “hybridization” of extremism facilitated by digital communications means that hierarchical extremist organizations like Islamic State and al‐Qaeda may not necessarily be the main ideologies attracting youth today (Jones and Dawson 2023). Understanding the ideological fragmentation and fluidity of youth radicalization is important to generating effective strategies for prevention and for deradicalization—“the process of cognitive change from criminal, radical, or extremist ideologies to a non‐criminal or moderate psychological state” (Koehler 2014, 420). Furthermore, much of the evidence on radicalization focuses on adult terrorists (Bronsard et al. 2022). Distilling findings about radicalization among school‐age youth is key to developing youth‐specific interventions.

### 1.2 Outcome—Youth Radicalization

The outcome of interest for the review is radicalization of school‐age youth. We propose a two‐part review that examines the prevalence of radicalization in Part 1 and risk/protective factors for radicalization in Part 2. With reference to the TPM of radicalization (McCauley and Moskalenko 2017) discussed above, measures of both radicalization of attitude and radicalization of behavior will be considered. Radicalization is generally measured through self‐reported and other‐reported changes in youth attitudes and beliefs toward the use of unlawful violence to effect societal or political change. Such measurement may range from validated scales that are administered to youth and children, to surveys and interviews that ask respondents to indicate identification with extremist views and/or willingness to advocate for, facilitate, or use unlawful violence. Examples of indicators of youth radicalization measured through these approaches might include agreement with statements on attitudinal scales that indicate likelihood of supporting or engaging in extremist acts, such as, “I would participate in a public protest against oppression of my group even if I thought the protest might turn violent” (Frounfelker et al. 2021). Interviews can collect more nuanced data on pathways from engagement to radicalization (Borum 2011). In addition, youth radicalization can be examined through analyzing online behaviors, such as engagement with extremist content, social media posts endorsing violence, or participation in radical forums (Awan 2017). Official records, such as police or school reports, can also be sources of indicators of youth radicalization behaviors.

The review will examine the prevalence of radicalization by different ideological groups such as those listed above, as identified in retrieved research documents (e.g., religiously motivated, far left and far right, ethno‐nationalist, racially and ethnically motivated, gender‐based, online conspiracy‐based). Because the review focuses on youth through high school/secondary school, generally age 18, we would expect that eligible studies will tend to focus on attitudes and beliefs versus behavior.

### 1.3 Predictive, Risk, or Protective Factors

In examining factors associated with radicalization, it is essential to distinguish among predictive, risk, and protective factors, as each type has a distinct implication for understanding pathways toward or away from radicalization. Predictive factors are characteristics that can be used to estimate the likelihood of an individual engaging in radicalization over time. To meet this criterion, a predictive factor must either precede the outcome of radicalization or be a stable, time‐independent trait (e.g., race/ethnicity, sex assigned at birth). Predictive factors are particularly valuable for intervention design, as they allow for early identification of individuals at greater risk. For example, prior exposure to violent ideologies could be a predictive factor if it reliably precedes radicalization incidents in a temporal sequence.

Research specifically focused on radicalization among youth indicates risk factors that align with those for adults, as well as factors that are specific to (or more influential) for youth. For example, in their review of the literature on radicalization among youth in Europe, Campelo et al. (2018) identify three categories of risk factors: individual, environmental, and societal. Individual risk factors for youth radicalization the authors identify include psychological vulnerabilities and identity formation issues. Environmental factors include family and peer networks of influence. Societal factors include group polarization and social exclusion, geopolitical and local political factors, and ideological narratives. Additional risk factors for youth radicalization identified by researchers that might be classified into the categories identified by Campelo et al. (2018) include cultural integration and intergenerational conflict [societal] (Lynch 2013), unemployment and educational disengagement [individual] (Neve et al. 2020), and activism and perceived in‐group superiority [societal, environmental] (Emmelkamp et al. 2020).

Protective factors for youth radicalization identified in the research coalesce around resilience and well‐being. For example, good health, connectedness, safe and supportive environment, positive parenting, educational success and employability, and agency and autonomy are components of adolescent well‐being that can protect against radicalization (Haghish et al. 2023). In addition, developing self‐control and coping skills, which are important aspects of resilience, may help reduce extremist attitudes (Nivette et al. 2017).

Risk factors are characteristics or conditions that increase an individual's likelihood of becoming radicalized. These can be demographic, psychological, social, or environmental in nature, such as socioeconomic disadvantage, exposure to extremist networks, or a history of discrimination. While risk factors can be predictive or correlational, predictive risk factors must meet temporal criteria (i.e., they precede the radicalization outcome) or be stable, time‐independent traits, similar to the distinction in predictive factors above.

Protective factors, on the other hand, are characteristics that reduce the likelihood of radicalization or mitigate its impact if radicalization tendencies are already present. Examples might include a strong support network, high resilience, or community engagement. Protective factors can counterbalance risk factors and are essential for understanding what conditions contribute to resilience against radicalization.

Correlates refer to factors that show an association with radicalization but do not establish causation or directionality. For example, low education levels or unemployment may be correlated with radicalization, but without establishing temporality or causal influence, they remain correlates. Correlates are informative for identifying patterns and generating hypotheses about potential risk or protective factors, but are not as useful for predicting radicalization or guiding interventions directly.

As outlined in previous systematic reviews examining factors associated with criminal recruitment and radicalization (Higginson et al. 2018; Calderoni et al. 2019), clearly distinguishing between these types of factors is critical for understanding their role in either contributing to or protecting against radicalization. Understanding the difference between predictive and risk/protective factors will guide this review in categorizing and interpreting findings, which ultimately may inform targeted prevention or intervention strategies.

Naturally, there are challenges with determining whether a risk/protective factor actually precedes an outcome like radicalization in time. We follow the practice of Higginson et al. (2018) and Calderoni et al. (2019) and will consider *predictors* to be factors from longitudinal studies that clearly preceded the measurement of the outcome (radicalization). Factors identified in cross‐sectional or other studies in which temporal ordering is in question will be considered *correlates* in our review. We will report these separately in our analyses.

We also note the interpretive challenge of whether a risk factor is simply the inverse of a protective factor. We do not assume this, and we will classify the factor as the authors of the primary study did, and not attempt to place our own interpretation on it, that is, if the primary authors analyzed education level as a protective factor, we would code it as such even if it could also serve as a risk factor.

### 1.4 Why the Factors Might Affect the Outcome

Factors such as those discussed above are delineated in several overlapping psycho‐social theoretical frameworks. For example, the 3N model (Szumowska et al. 2020) suggests that individuals may radicalize due to a *need* for significance, a *narrative* that justifies extremist actions, and a *network* that provides support for these actions. Relative deprivation theory (Gurr 1970) can be applied to factors influencing radicalization, as individuals or groups who feel deprived compared to others around them may develop grievances that can lead to radicalization. Social learning theory (Bandura 1977) holds that behaviors are learned through observing and imitating others, especially authority figures or admired peers. Thus, youth may be influenced by role models—whether in person or online—who promote extremist ideologies. Similarly, social identity theory (Tajfel and Turner, 1979) suggests that people derive a sense of identity and self‐worth from their social group memberships. Identifying strongly with an in‐group can lead to the rejection or demonization of out‐groups, potentially fostering extremist attitudes. While each of these frameworks offers unique insights into specific factors that can influence youth extremism, our review will encompass the range of factors identified in the included studies.

As mentioned above, McCauley and Moskalenko's (2017) TPM of cognitive and behavioral radicalization presents a framework for understanding pathways by which risk and protective factors might affect youth radicalization outcomes. The model holds beliefs and actions as distinct, with the cognitive pyramid reflecting the escalation of ideological commitment from sympathizing with a cause to justifying the use of violence but not acting on those beliefs, to feeling a moral obligation to support violent actions to promote the cause, and the behavioral pyramid showing a progression from non‐violent to violent action. An aim of the review is to elucidate how different individual, environmental, and societal factors influence youth progression toward or away from extremism of opinion or extremism of behavior.

For example, ideological exposure in a youth's environment could move their attitudes along the cognitive spectrum from neutral to sympathetic. Similarly, social bonds with individuals who engage in radical actions could move youth up the behavioral pyramid from non‐violent action in support of a cause to violent behavior. On the other hand, environmental exposure to diverse perspectives or educational opportunities could move youth's attitudes away from extremist views and behaviors.

Integrating the TPM with findings about youth radicalization risk/protective factors presents opportunities for assessing vulnerabilities and targeting interventions. For example, youth moving up the cognitive pyramid due to ideological societal narratives could benefit from interventions employing counternarrative programs. Youth on the action pyramid due to social exclusion might benefit from targeted education or employment opportunities. In addition, understanding how certain risk and protective factors might influence movement between belief and action could inform interventions to prevent youth from moving from the beliefs to the action pyramid. Assessing where youth fall on the beliefs/action spectrum could also indicate where law enforcement deterrence might become necessary.

### 1.5 Why It Is Important to Do the Review

The two parts of this review are prevalence and risk/protective factors. Both provide value added for the prevention of violent extremism. In Part 1, systematically reviewing prevalence data provides more stable estimates of the scope and scale of youth radicalization. This can help society allocate resources appropriately and tailor programs to the needs of at‐risk groups. Examining prevalence rates, for example, over time, allows us to determine if the problem is increasing or decreasing. Breaking this down by subgroups, if so analyzed by primary authors, will also permit us to see whether the problem is more significant, for example, among certain ideologies.

In Part 2, understanding the factors that may influence youth to move toward extremism or not is a major step in knowing what to do to stop it (Harpviken 2020). How to appropriately assess and intervene with these radicalized youth is a nascent field, and biased assumptions about the causes and manifestation of youth radicalization can result in inefficient or ineffective policy and program responses (Bronsard et al. 2022). Effective assessment and responses will reflect what is known about risk factors for youth, as well as knowledge of how youth exercise resilience and coping strategies and how these can best be supported. Developing a comprehensive understanding of these factors would facilitate efforts to decrease the risks and increase protective factors through prevention and deradicalization policy and programming aimed specifically at youth.

While existing research syntheses (e.g., Sarma et al. 2022; Zych and Nasaescu 2022; Wolfowicz et al. 2021) explore radicalization prevalence and risk and protective factors in general populations, and Emmelkamp et al. (2020) examine risk (but not protective) factors for radicalization among juveniles, we are not aware of a systematic review that addresses prevalence of youth radicalization. Understanding what is known about the scope and scale of youth radicalization across different regions and contexts and identifying radicalization risk and protective factors for youth can inform targeted prevention and intervention strategies, as well as identify gaps in the knowledge base and areas for further research. None of the prior reviews examine youth of this specific age range (generally, 5–17 years of age), but include overlapping samples of young adults (e.g., 18–21).

## 2 Objectives

This two‐part systematic review will synthesize published and unpublished scientific literature on (1) the prevalence of and (2) the risk and protective factors for school‐aged youth radicalization. In the nascent field of research on radicalized youth, a priority of the review is to examine the breadth of empirical evidence on prevalence and risk and protective factors, and to identify gaps and opportunities for further research, rather than to address existing discrepancies in the field.

Our research questions are:

Part 1: Prevalence

1. What is the prevalence of radicalization among school‐aged youth?
2. How does the prevalence of radicalization of school‐aged youth vary over time and by type of radical ideology (e.g., religiously motivated, far left and far right, ethno‐nationalist, racially and ethnically motivated, gender‐based, online conspiracy‐based)?

Part 2: Risk and Protective Factors

1. What are the cognitive and behavioral risk and protective factors for school‐aged youth radicalization?
2. How do cognitive and behavioral risk and protective factors for youth radicalization vary by type of radical ideology (e.g., religiously motivated, far left and far right, ethno‐nationalist, racially and ethnically motivated, gender‐based, online conspiracy‐based)?

## 3 Methods

### 3.1 Criteria for Considering Studies for This Review

#### 3.1.1 Types of Studies

The review will search for and screen for inclusion studies with outcomes that fall under the two parts of the review—prevalence and risk/protective factors—for youth radicalization. As detailed below, the search strategy is calibrated to efficiently identify studies for both parts of the review. We will not limit studies by publication year, type of publication (gray literature publications will be included), location, or language. Studies using languages other than English will be translated into English via ChatGPT.

**Part 1:** Prevalence research will include cross‐sectional studies that examine the prevalence of radicalization within a sample. It will also include cohort studies that report the prevalence of the outcome in the sample for at least one follow‐up interval. To be eligible for inclusion, studies must provide empirical findings with sufficient methodological detail—including a description of sampling procedures, data collection methods, measurement details, and statistical analysis—to allow for replicability and enable computation of effect sizes. They must report at least one prevalence estimate for radicalization (e.g., 4% of the sample self‐reported radicalized views). These criteria exclude studies lacking transparency in reporting or studies that use qualitative methods alone (e.g., case studies or unstructured interviews), as these do not permit computation of standardized effect sizes or comparisons across studies. Similarly, purely theoretical or conceptual papers, which lack primary empirical data, are also excluded. Both published and unpublished studies are potentially eligible for inclusion if they meet the above criteria.

**Part 2:** Studies will be included for the second part of the review if they provide empirical data on the relation between any possible risk/protective factor and school‐aged youth radicalization. These can be correlational studies, or studies that compare groups, e.g., with and without the risk/protective factor, or radicalized versus non‐radicalized. To be eligible for inclusion, studies must provide empirical findings with sufficient methodological detail—including a description of sampling procedures, data collection methods, measurement details, and statistical analysis—to allow for replicability and enable computation of effect sizes. Studies must report the minimum information that would allow for the computation of an effect size, such as a correlation coefficient. These criteria exclude studies lacking transparency in reporting or studies that use qualitative methods alone (e.g., case studies or unstructured interviews), as these do not permit computation of standardized effect sizes or comparisons across studies. Similarly, purely theoretical or conceptual papers, which lack primary empirical data, are also excluded. Both published and unpublished studies are potentially eligible for inclusion if they meet the above criteria.

#### 3.1.2 Types of Participants

Both parts 1 and 2 of the review will include research conducted on youth up to and including age 18. We note that for school samples, older students in high school (e.g., 19) are usually included in broad surveys, so our samples may include a small number of older youth. We will accept studies that use overlapping samples if there is evidence presented that most of the youth (at least 80%) are under age 19 or that the prevalence or risk/protective data can be isolated for such youth. There are no restrictions for participants in terms of ethnicity, gender, SES, location, or any other characteristics.

#### 3.1.3 Types of Outcome Measures

Outcome measures will include the two tiers discussed by Wolfowicz et al. (2021): cognitive and behavioral. This review will collect data on cognitive radicalization, which would include measures of self‐reported and other‐reported attitudes and beliefs by youth toward the use of unlawful violence to effect societal or political change. This can include the use of validated instruments or broad surveys that ask items relevant to radicalization. For example, scales such as the Radicalism Intention Scale (RIS) collect self‐reported information on a person's willingness to support or engage in violent and illegal actions in the name of a group or ideology.

Behavioral radicalization would be outcomes of actual extremist behavior (violence or threat of violence) that could be measured through official reports (e.g., police or court records) or school or community reporting. Although extremist violence by school‐aged youth seems very unlikely, studies that collected behavioral data (e.g., attending and signing up to be a member of a hate group) will be included.

No secondary outcomes are anticipated for this review. We also anticipate that the radicalization outcomes for Part I and Part II will be the same.

#### 3.1.4 Types of Predictive, Risk, or Protective Factors

For Part I, we anticipate estimating the overall prevalence across studies. Potentially, if information and sample permits, we will examine subgroups such as prevalence for specific regions of the world or among specific ideological groups. We caution, however, that Wolfowicz et al. (2021), in their much larger study of risk and protective factors for radicalization, did not have enough information to reliably perform a single subgroup analysis.

Research specifically focused on radicalization among youth suggests risk factors that align with those for adults, as well as factors that are specific to or more influential for youth. For example, in their review of the literature on radicalization among youth in Europe, Campelo et al. (2018) identify a range of risk factors falling into three categories: individual, environmental, and societal. Individual risk factors for youth radicalization include psychological vulnerabilities and identity formation issues. Environmental factors include family and peer networks of influence. Societal factors include group polarization and social exclusion, geopolitical and local political factors, and ideological narratives. Additional risk factors for youth radicalization identified by researchers include cultural integration and intergenerational conflict (Lynch 2013), unemployment and educational disengagement (Neve et al. 2020), and activism and perceived in‐group superiority (Emmelkamp et al. 2020).

Protective factors for youth radicalization identified in the research coalesce around resilience and well‐being. For example, good health, connectedness, safe and supportive environment, positive parenting, educational success and employability, and agency and autonomy are components of adolescent well‐being that can protect against radicalization (Haghish et al. 2023). In addition, developing self‐control and coping skills, which are important aspects of resilience, may help reduce extremist attitudes (Nivette et al. 2017).

Although this section provides examples of factors with a prior research base, our review will include any factors that studies examine for a relationship to radicalization.

#### 3.1.5 Types of Settings

Relevant studies conducted with school‐age youth in all types of settings (school, community, youth programs and centers, religious institutions, youth correctional facilities, online spaces, etc.) will be screened for inclusion.

### 3.2 Search Methods for Identification of Studies

#### 3.2.1 Electronic Searches

In collaboration with the author team, an experienced information specialist has designed a core search strategy composed of keyword search terms and subject headings to identify studies relevant to Parts 1 and 2 of the review. This core strategy applied to PsycINFO is provided in Table 1. This will be translated for the remaining databases (Table 2) using the appropriate syntax for each platform and database. All searches will be run across the Title, Abstract, Author Supplied Keywords, and Subject/Indexing search fields (where available). We will not limit the searches by publication date or language.

**Table 1:** Core search strategy.

APA PsycInfo <1806 to August 2024 Week 2>	
1	(school age 6 12 yrs or adolescence 13 17 yrs).ag.
2	(adolesc* or boy* or child* or girl* or juvenile* or minor or minors or preadolesc* or prepubescen* or puberty or pubescen* or teen* or young* or “young people” or youth*).ti,ab,id.
3	(“elementary school*” or “high school*” or “junior high” or “middle school*” or “primary school*” or pupil* or “secondary school*” or “school age*” or “school‐age*” or student*).ti,ab,id.
4	1 or 2 or 3
5	extremism/
6	exp terrorism/
7	radical movements/
8	(extreme‐left or extreme‐right or “extreme violen*” or extremism or extremist or extremists).ti,ab,id.
9	terroris*.ti,ab,id.
10	(anarch* or bioterror* or far‐left or far‐right or fighter* or “foreign agent*” or “foreign fighter*” or incel or incels or indoctrinat* or islamis* or jihadi* or lone or loner* or militant* or militia* or sympathiser* or sympathizer*).ti,ab,id.
11	(nationalis* or neo‐nazi* or radicali* or radicals or salafi* or “violent ideolog*” or “white supremacis*”).ti,ab,id.
12	(radical* adj3 (belief* or group* or ideolog* or left* or movement* or organisation* or organization* or right* or theolog*)).ti,ab,id.
13	5 or 6 or 7 or 8 or 9 or 10 or 11 or 12
14	(frequency or incidence or prevalent* or rate or rates).ti,ab,id.
15	risk factors/
16	(risk* adj3 factor*).ti,ab,id.
17	((risk* or factor*) adj5 (causal or cause or caused or causing or correlat* or determin* or predict* or putative*)).ti,ab,id.
18	protective factors/
19	(protect* adj3 factor*).ti,ab,id.
20	((protect* or factor*) adj5 (causal or cause or caused or causing or correlat* or determin* or predict* or putative*)).ti,ab,id.
21	15 or 16 or 17 or 18 or 19 or 20
22	4 and 13 and (14 or 21)
23	animal.po.
24	exp animals/or animal models/or exp animal research/or exp primates/
25	23 or 24
26	human.po.
27	25 not 26
28	22 not 27

**Table 2:** Databases and platforms.

Database	Platform
Australian Criminology Database (CINCH)	Informit
APA PsycEXTRA	Ovid
APA PsycINFO	Ovid
MEDLINE	Ovid
Applied Social Sciences Index & Abstracts (ASSIA)	ProQuest
Dissertations & Theses Global	ProQuest
Educational Resources Information Center (ERIC)	ProQuest
International Bibliography of the Social Sciences (IBSS)	ProQuest
Social Services Abstracts	ProQuest
Sociological Abstracts	ProQuest
Sociology Database	ProQuest
Conference Proceedings Citation Index ‐ Social Sciences & Humanities (CPCI‐SSH)	Web of Science
Book Citation Index – Social Sciences & Humanities	Web of Science
Emerging Sources Citation Index (ESCI)	Web of Science
Social Science Citation Index (SSCI)	Web of Science
Criminal Justice Abstracts	EBSCO
International Political Science Abstracts	EBSCO
Scopus	Elsevier
Social Science Research Network (SSRN)	www.SSRN.com

#### 3.2.2 Searching Other Resources

We will also search the websites of the following organizations for relevant gray literature and will reach out to experts in the area of youth radicalization at these and other organizations to identify relevant studies. We will also reach out to the authors of the included studies.

- Australian Institute of Criminology (https://www.aic.gov.au).
- Center for Counter‐Terrorism Coordination (https://www.homeaffairs.gov.au/).
- U.S. Department of Homeland Security (https://www.dhs.gov).
- European Expert Network on Terrorism Issues (https://www.european-enet.org).
- Five Research and Development (5RD) Countering Violent Extremism Network (https://www.dhs.gov/archive/science-and-technology/terrorism-prevention-resources).
- Global Research Network on Terrorism and Technology (https://rusi.org/explore-our-research/projects/global-research-network-terrorism-and-technology).
- Global Terrorism Research Center (https://www.monash.edu/arts/social-sciences/gtrec).
- Hedayah (https://hedayah.com).
- UK Home Office (https://www.gov.uk/government/organisations/home-office).
- National Consortium for the Study of Terrorism and Responses to Terrorism‐START (https://www.start.umd.edu).
- Public Safety Canada (https://www.publicsafety.gc.ca).
- EU Radicalisation Awareness Network (https://home-affairs.ec.europa.eu/networks/radicalisation-awareness-network-ran_en).
- Radicalisation Research (https://radicalisationresearch.org).
- Terrorism Research Center (https://terrorismresearch.uark.edu).
- Vox‐Pol Network of Excellence‐NoE (https://voxpol.eu).
- National Criminal Justice Reference Service‐NJCRS (https://www.ojp.gov/ncjrs).
- Center for Research and Evidence on Security Threats (CREST).
- National Counterterrorism Innovation, Technology, and Education (NCITE) Center.

In addition, we will harvest relevant reviews and all included studies and will conduct a forward citation search of all included articles in Google Scholar. We will also conduct electronic searches of the table of contents and abstracts from relevant journals to identify any studies published in the last 2 years that may not yet be indexed online. These journals are:

- *Journal for Deradicalization* (https://www.scopus.com/sourceid/21100967061).
- *Journal for the Study of Radicalism* (https://www.jstor.org/journal/jstudradi).
- *Perspectives on Terrorism* (https://www.jstor.org/journal/persponterr).
- *Studies in Conflict & Terrorism* (https://www.tandfonline.com/journals/uter20).

### 3.3 Data Collection and Analysis

#### 3.3.1 Selection of Studies

As outlined below, we will first review all citations (titles and abstracts) to determine if the cited studies should proceed to a second screening as a potentially eligible study. During the second screening, we will retrieve full text documents and apply the screening questions to determine whether studies are eligible for full review. One person (Morgan) will independently do the initial screening and identify any challenging studies for discussion and resolution (Petrosino and Morgan).

**First Screening (titles and abstracts)**

1. Population & Sample
  - Does the study population consist of primary or secondary school‐aged youth?
2. Study Focus and Outcome Types
  - Does the study assess radicalization among youth?
3. Study Design & Methodology
  - Does the study include quantitative findings on youth radicalization?

**Second Screening (full text)**

1. Study Design & Methodology
  - Does the study include quantitative findings on radicalization prevalence or on the relationship between a risk/protective factor and youth radicalization?
  - Are the study's empirical findings reported in enough detail to replicate methods and sampling?
  - Are the study's empirical findings reported in enough detail to compute an effect size?
    ∘ If the answer is “no” to just the final two questions, we will put the study in the “to be determined” (TBD) pile and follow up with study authors to see if we can retrieve the data.
  - For prevalence:
    ∘ Is the study a cross‐sectional or cohort design that provides prevalence data on radicalization in the sample?
  - For risk/protective factors:
    ∘ Does the study examine and report on a potential predictor, correlate, or risk/protective factor in relation to youth radicalization?

Bibliographic details from included and excluded studies will be entered into SmartSheet for recording. All searches will be tracked to allow us to provide details on the pipeline of retrieved citations to our final sample. We will complete a PRISMA flow diagram to display the results of the search and screening process and will fully describe these processes and results in the narrative, as well.

#### 3.3.2 Data Extraction and Management

The information for each study will be extracted using a specially designed two‐part coding instrument. Basic information (e.g., authors, year of publication, type of publication) will be collected from all studies. Then the instrument will ask if the study is focused on prevalence or risk/protective factors. Depending on the response, the coder will skip to more specific items about that specific part of the review. Information will be extracted relevant to

- Study design (e.g., cohort or cross sectional).
- Populations and sampling.
- Comparison groups, if relevant.
- Data sources and types.
- Measures.
- Outcomes.

Petrosino and Morgan will each code two prevalence studies and two risk/protective studies independently. Then they will discuss any challenges or conflicts. The instrument will be revised accordingly. Petrosino will proceed with coding prevalence studies, and Morgan will focus on the risk/protective studies. Based on our initial passes through the literature, it is likely there will be far more risk/protective studies than prevalence studies, so Petrosino will code those reports that fit into both reviews (to reduce coding load on Morgan). They will meet to discuss any further challenges to coding and resolve difficulties. If items need to be changed or dropped, we will note it in our final report.

#### 3.3.3 Assessment of Risk of Bias in Included Studies

Similar to Zych and Nasaescu (2022), we do not plan to eliminate eligible studies due to quality but will take potential bias into account. Including a table of potential bias of each included study will allow readers to assess the reliability of results.

For studies of prevalence, we will code each study using RoB‐PrevMH (Tobias et al. 2023) to assess the risk of bias in prevalence studies. This is a three‐item instrument that has had successful reliability rates, high marks for simplicity, and strong consistency with items identified from previous such instruments in reviews of prevalence studies. This instrument extracts data from each study about sampling, response, and outcomes (see coding instrument in Appendix for more detail on items).

For risk/protective factors, we will code the quality of studies using the Cambridge Quality Checklists for correlations and for risk factors (Murray et al. 2009). These checklists assess each study on the basis of sample size and representativeness, response rates, reliability of measurement for the outcome and the risk/protective factor, and type of design used (e.g., longitudinal). See the coding instrument in Appendix for more detail on items.

As aforementioned, before beginning the review, both reviewers (Morgan and Petrosino) will independently review two eligible studies in Part 1 and Part 2, respectively, to check reliability and identify issues with the instrument that require discussion. This includes assessment of the risk of bias. Following calibration activities to resolve challenges, Petrosino (for prevalence) and Morgan (for risk/protective) will proceed with the extraction of data for all studies in the sample. Challenges that are identified in this data extraction with coding any of the individual studies, including risk of bias, will be brought to a joint meeting for discussion and resolution.

As mentioned below, risk of bias will be included in a sensitivity analysis to better understand if it plays a role in observed study estimates.

#### 3.3.4 Measures of Predictive, Risk, or Protective Factors

Measurement of risk/protective factors will largely come from either validated instruments or broader surveys and questionnaires that ask youth to self‐report radicalization. Less common will be ratings or assessments by teachers, parents, clinicians, or others. It is possible that studies that use official or administrative records from an agency that measure risk/protective factors may also be included in our sample.

#### 3.3.5 Measures of the Outcome

Our assumption is that most studies will measure the prevalence of cognitive radicalization by estimating it from self‐reported surveys of students. Other measurements will come from validated instruments administered to students that may include a score that indicates the existence of radicalization, and ratings by a significant other (e.g., by teachers or clinicians). The prevalence of behavioral radicalization will be measured by official records documenting an extremist act, or via surveys and interviews that ask youth to self‐report radicalized behavior. We will include studies that use non‐validated instruments but will use sensitivity analyses to determine if results from such studies impact conclusions from meta‐analyses.

#### 3.3.6 Unit of Analysis Issues

We anticipate that our review will include only studies that have the individual as the unit of analysis. However, in the unlikely event that we have primary studies that included larger aggregate units such as schools as the unit of analysis (e.g., a survey of prevalence in the context of a group level RCT), we will analyze group‐level studies separately, following Fisher et al. (2023).

We reiterate that all of our meta‐analyses will be held distinct by each factor and each outcome; we will not be lumping them all into one global meta‐analysis.

For the risk/protective factors studies, we anticipate just one effect size per factor from each study. This was what happened in a large systematic review of risk/protective factors for cognitive and behavioral radicalization (Wolfowicz et al. 2021).

We will code all analyses including those that report both adjusted and unadjusted estimates for the same factor. We will report on adjusted and unadjusted effects separately, for each risk factor. For cohort studies with the same or overlapping samples, we will pool the effects across cohorts.

#### 3.3.7 Dealing With Missing Data

For studies with missing data, we will reach out to the study authors to request additional information. At minimum, if a study includes a prevalence estimate, it will be included in the prevalence, and if it includes at least one estimate of a relationship between a factor and an outcome, it will be included in the risk/protective factor review. However, if data are missing for other variables (e.g., geography), listwise deletion will be used and that study will be excluded from that specific analysis. We will include a table as an appendix that summarizes descriptive data and provides the frequency of missing values for each study characteristic.

#### 3.3.8 Assessment of Heterogeneity

We will assess heterogeneity of studies for both Part 1 and Part 2. We will report results from the Cochran's *Q* Test and *I* ^2^ analysis to statistically assess the presence of heterogeneity, but will note our caution if the sample of review studies included is small. The *Q* statistic indicates heterogeneity if it is not significant, and *I* ^2^ is used to indicate the degree of heterogeneity. Following Wolfowicz et al. (2021), we will interpret that an *I* ^2^ score of greater than 75 will indicate high heterogeneity (and following, very low scores between 1 and 24; low scores between 25 and 49; and moderate scores between 50 and 74). An absence of heterogeneity will be indicated by zero.

#### 3.3.9 Assessment of Reporting Biases

We agree with Caicedo‐Roa et al. (2020) that the best method for handling bias is to prevent it in the first place. We aim to limit reporting or publication bias by a robust and comprehensive search strategy that includes gray literature searches. Similar to their review on risk factors for femicide, if we have over 10 studies included in either part of this review, we will report a funnel plot on the relationship between publication and the radicalization outcome. The clearest way to identify the potential of publication bias is to conduct a sensitivity analysis to examine results between published and unpublished studies, provided the sample of studies includes both (which we anticipate). For Part 1, we will examine whether prevalence estimates differ for published versus unpublished studies. Similarly, for Part II, we will compare published versus unpublished studies for each risk/protective factor included in the analysis. We propose that this will be much more effective and efficient than examining the symmetry of a funnel plot for each risk/protective factor.

#### 3.3.10 Data Synthesis

To perform synthesis of prevalence studies, we will report a pooled prevalence estimate. Similar to Yon et al. (2017), we will use the proportion of youth experiencing radicalization for each study. Using procedures reported in Yon et al., we will perform a logit transformation, and the pooled estimates will be calculated using the DerSimonian and Laird method (1986).

For the risk/protective factors, we will use Pearson's correlation coefficient (*r*) as our effect size. We will convert effect sizes from reports that did not report Pearson's *r* using equations from Lipsey and Wilson (2001). Following Wolfowicz et al. (2021), we will conduct a separate meta‐analysis for each identified factor, presenting the results as *r* correlations with 95% confidence intervals.

The data will be entered into Stata statistical software. The Stata meta‐analysis syntax (“meta”) allows one to conduct meta‐analysis of all types. We will use Stata to statistically combine results from prevalence studies in one meta‐analysis and then from the risk/protective factors in a series of meta‐analyses as described above. We will assume random effects models are assumed, given that we expect to find studies reported around the world, including the United States, Canada, different countries in Europe, and Australia.

We will provide tables and detailed narrative summaries of the characteristics of the included studies, including designs, settings, samples, measures, prevalence outcomes, and risk and protective factors identified in the studies. We will also discuss the risk of bias in included studies and provide a summary of excluded studies. We will then outline and discuss the results of the synthesis of prevalence, followed by a discussion of the risk and protective factors. We will categorize and discuss the risk and protective factors by individual, environmental, and societal factors, along with sub‐categories that emerge from the findings.

#### 3.3.11 Subgroup Analysis and Investigation of Heterogeneity

Statistical analyses of subgroups/moderators will depend on the number of studies that are included in our prevalence and risk/protective analyses. Following Wolfowicz et al. (2021), if we achieve over 10 studies in Part 1 or Part 2 for any of these analyses, we will examine how study estimates differ across the type of underlying radicalization (political, religious, hate), the type of outcome (cognitive vs. behavioral), geography, and the methodological design used (e.g., cohort/longitudinal vs. cross sectional). We will conduct simple analyses, if possible, using Cochran's *Q* and *I* ^2^ to indicate if a subgroup result differs by chance or not, and what the strength of the relationship is.

#### 3.3.12 Sensitivity Analysis

Our plan is to conduct sensitivity analyses to assess the role of potential bias in findings. Following the methods proposed by Caicedo‐Roa et al. (2020) in their review of risk factors for femicide, we will perform sensitivity analysis to see how studies rated to be at “high” risk of bias differ from those rated as having a “low” risk of bias. This will be conducted for the prevalence analysis and the risk/protective factor analysis. We will also examine whether the inclusion of studies with unvalidated instruments changes findings by analyzing results with and without these studies. To examine publication bias, we propose sensitivity analyses of findings from published versus unpublished reports.

## Author Contributions

- Content: Anthony Petrosino and Claire Morgan
- Systematic review methods: Anthony Petrosino and Claire Morgan
- Information retrieval: Anthony Petrosino and Claire Morgan

Anthony Petrosino has over 30 years of experience in conducting systematic reviews and meta‐analyses focused on different topics related to justice, violence, and international development. He was a Founding Member of the Campbell Collaboration and has successfully led and published four prior reviews for the Campbell Collaboration. Claire Morgan has co‐authored several prior systematic reviews, including a Campbell Collaboration review of research on the effects of school enrollment programs in developing nations.

## Conflicts of Interest

The authors declare no conflicts of interest.

## Preliminary Timeframe

We plan to submit the first draft of the review by in summer, 2025. We will submit a final draft by fall, 2025.

## Plans for Updating the Review

Petrosino and Morgan will plan to update the final review within 3 years of publication, depending on available resources.

## Supporting information


**Appendix. Instrument for data extraction of primary studies**.

## Endnotes

1 Terrorism is defined by the U.S. Department of Homeland Security (2017) as “Involving acts dangerous to human life that are a violation of the criminal laws of the United States or of any State; Appearing to be intended to: Intimidate or coerce a civilian population; Influence the policy of government by intimidation or coercion; or Affect the conduct of a government by mass destruction, assassination, or kidnapping.”

## References

[cl270041-bib-0031] Awan, I. 2017. “Cyber‐Extremism: ISIS and the Power of Social Media.” Society 54, no. 2: 138–149. 10.1007/s12115-017-0114-0.

[cl270041-bib-0032] Bandura, A. 1977. “Self‐Efficacy: Toward a Unifying Theory of Behavioral Change.” Psychological Review 84, no. 2: 191–215. 847061 10.1037//0033-295x.84.2.191

[cl270041-bib-0033] Borum, R. 2011. “Radicalization Into Violent Extremism I: A Review of Social Science Theories.” Journal of Strategic Security 4: 7–36. 10.5038/1944-0472.4.4.1.

[cl270041-bib-0001] Bronsard, G. , D. Cohen , I. Diallo , et al. 2022. “Adolescents Engaged in Radicalisation and Terrorism: A Dimensional and Categorical Assessment.” Frontiers in Psychiatry 12 (January): 774063. 10.3389/fpsyt.2021.774063. 35095595 PMC8795583

[cl270041-bib-0034] Caicedo‐Roa, M. , T. D. A. Pereira , and R. C. Cordeiro . 2020. “PROTOCOL: Risk Factors for Femicide.” Campbell Systematic Reviews 16, no. 4: e1123. 10.1002/cl2.1123. 37016606 PMC8356346

[cl270041-bib-0002] Calderoni, F. , E. Superchi , T. Comunale , G. M. Campedelli , M. Marchesi , and N. Frualdo . 2019. “PROTOCOL: Organised Crime Groups: A Systematic Review of Individual‐Level Risk Factors Related to Recruitment.” Campbell Systematic Reviews 15 (June): 1–2. 10.1002/cl2.1022 PMC8356535 37131479

[cl270041-bib-0003] Campelo, N. , A. Oppetit , F. Neau , D. Cohen , and G. Bronsard . 2018 Aug. “Who Are the European Youths Willing to Engage in Radicalisation? A Multidisciplinary Review of Their Psychological and Social Profiles.” European Psychiatry 52: 1–14. 10.1016/j.eurpsy.2018.03.001. 29614388

[cl270041-bib-0035] Canadian Security Intelligence Service . n.d. Mobilization to Violence (Terrorism) Research: Key Findings. Author.

[cl270041-bib-0036] Council of the European Union . 2005. “The European Union Counter‐Terrorism Strategy.” November 30. https://www.refworld.org/policy/legalguidance/council/2005/en/61243.

[cl270041-bib-0007] Emmelkamp, J. , J. J. Asscher , I. B. Wissink , and G. J. J. M. Stams . 2020. “Risk Factors for (Violent) Radicalization in Juveniles: A Multilevel Meta‐Analysis.” Aggression and Violent Behavior 55: 101489. 10.1016/j.avb.2020.101489.

[cl270041-bib-0037] Erdemandi, M. , E. Savoia , and M. J. Williams . 2024. “Assessing the Effectiveness of Programs to Prevent and Counter Violent Extremism.” NIJ Journal: 285. https://nij.ojp.gov/topics/articles/assessing-effectiveness-programs-prevent-and-counter-violent-extremism.

[cl270041-bib-0038] Federal Bureau of Investigation . 2016. Preventing Violent Extremism in Schools. Author.

[cl270041-bib-0008] Federal Bureau of Investigation & Department of Homeland Security . 2023. Strategic Intelligence Assessment and Data on Domestic Terrorism. Accessed on May 3, 2024. https://www.fbi.gov/file-repository/fbi-dhs-domestic-terrorism-strategic-report-2023.pdf/view.

[cl270041-bib-0010] Fisher, B. W. , A. Petrosino , H. Sutherland , et al. 2023. “School‐Based Law Enforcement Strategies to Reduce Crime, Increase Perceptions of Safety, and Improve Learning Outcomes in Primary and Secondary Schools: A Systematic Review.” Campbell Systematic Reviews 19: e1360. 10.1002/cl2.1360. 38024781 PMC10630714

[cl270041-bib-0039] Frounfelker, R. L. , T. Frissen , D. Miconi , et al. 2021. “Transnational Evaluation of the Sympathy for Violent Radicalization Scale: Measuring Population Attitudes Toward Violent Radicalization in two Countries.” Transcultural Psychiatry 58, no. 5: 669–682. 10.1177/13634615211000550. 33990162 PMC8733345

[cl270041-bib-0040] Gurr, T. 1970. Why Men Rebel. Princeton University Press.

[cl270041-bib-0011] Haghish, E. F. , M. Obaidi , T. Strømme , T. Bjørgo , and C. Grønnerød . 2023. “Mental Health, Well‐Being, and Adolescent Extremism: A Machine Learning Study on Risk and Protective Factors.” Research on Child and Adolescent Psychopathology 51, no. 11 (November): 1699–1714. 10.1007/s10802-023-01105-5. 37535227 PMC10627959

[cl270041-bib-0012] Harpviken, A. N. 2020. “Psychological Vulnerabilities and Extremism Among Western Youth: A Literature Review.” Adolescent Research Review 5, no. 1: 1–26. 10.1007/s40894-019-00108-y.

[cl270041-bib-0013] Higginson, A. , K. Benier , Y. Shenderovich , L. Bedford , L. Mazerolle , and J. Murray . 2018. “Factors Associated With Youth Gang Membership in Low‐ and Middle‐Income Countries: A Systematic Review.” Campbell Systematic Reviews 14: 1–128. 10.4073/csr.2018.11. PMC8427992 37131383

[cl270041-bib-0041] Horgan, J. 2008. “From Profiles to Pathways and Roots to Routes: Perspectives From Psychology on Radicalization Into Terrorism.” ANNALS of the American Academy of Political and Social Science 618, no. 1: 80–94. 10.1177/0002716208317539.

[cl270041-bib-0014] Jones, D. A. , and L. L. Dawson . 2023. “Re‐Examining the Explanations of Convert Radicalization in Salafi‐Jihadist Terrorism With Evidence From Canada.” Behavioral Sciences of Terrorism and Political Aggression 15, no. 2: 246–273.

[cl270041-bib-0015] Koehler, D. 2014. “Deradicalization. Chapter 35.” The Routledge International Handbook on Hate Crime (1st ed.), edited by N. Hall , Routledge.

[cl270041-bib-0016] Lipsey, M. W. , and D. W. Wilson . 2001. Practical Meta‐Analysis. Sage.

[cl270041-bib-0017] Lynch, O. 2013. “British Muslim Youth: Radicalisation, Terrorism and the Construction of the “Other”.” Critical Studies on Terrorism 6, no. 2: 241–261.

[cl270041-bib-0019] McCauley, C. , and S. Moskalenko . 2017. “Understanding Political Radicalization: The Two‐Pyramids Model.” American Psychologist 72, no. 3: 205–216. 10.1037/amp0000062. 28383974

[cl270041-bib-0021] Murray, J. , D. P. Farrington , and M. P. Eisner . 2009. “Drawing Conclusions About Causes From Systematic Reviews of Risk Factors: The Cambridge Quality Checklists.” Journal of Experimental Criminology 5, no. 1: 1–23. 10.1007/s11292-008-9066-0.

[cl270041-bib-0022] Neve, R. J. , F. M. Weerman , S. Eris , and J. W. van Prooijen . 2020. “Radicalisation and Travelling to Syria Among Delinquent Youths: A Case Study From the Netherlands.” Journal for Deradicalization 22: 249–286.

[cl270041-bib-0023] Nivette, A. , M. Eisner , and D. Ribeaud . 2017. “Developmental Predictors of Violent Extremist Attitudes: A Test of General Strain Theory.” Journal of Research in Crime and Delinquency 54, no. 6: 755–790. 10.1177/0022427817699035.

[cl270041-bib-0024] Sarma, K. M. , S. L. Carthy , and K. M. Cox . 2022. “Mental Disorder, Psychological Problems and Terrorist Behaviour: A Systematic Review and Meta‐Analysis.” Campbell Systematic Reviews 18: e1268. 10.1002/cl2.1268. 36913225 PMC9364674

[cl270041-bib-0042] Szumowska, E. , A. Czernatowicz‐Kukuczka , M. Kossowska , S. Król , and A. Kruglanski . 2020. “Truth and Significance: A 3N Model (Needs, Narratives, Networks) Perspective on Religion.” In The Science of Religion, Spirituality, and Existentialism, edited by K. E. Vail and C. Routledge , 225–242. Academic Press.

[cl270041-bib-0043] Tajfel, H. , and J. C. Turner . 1979. “An Integrative Theory of Intergroup Conflict.” In The Social Psychology of Intergroup Relations, edited by W. G. Austin and S. Worchel , 33–47. Brooks/Cole.

[cl270041-bib-0025] Tobias, T. , D. Buitrago‐Garcia , N. L. Peter , et al. 2023. “Tool to Assess Risk of Bias in Studies Estimating the Prevalence of Mental Health Disorders (RoB‐PrevMH).” BMJ Mental Health 26, no. 1 (October): e300694. 10.1136/bmjment-2023-300694. PMC10619100 37899074

[cl270041-bib-0026] United Nations . 2016. Handbook on the Management of Violent and Extremist Prisoners and the Prevention of Radicalization to Violence in Prisons. https://www.unodc.org/pdf/criminal_justice/Handbook_on_VEPs.pdf.

[cl270041-bib-0027] U.S. Department of Homeland Security . 2017. DHS Lexicon Terms and Definitions. https://www.dhs.gov/sites/default/files/publications/18_0116_MGMT_DHS-Lexicon.pdf.

[cl270041-bib-0028] Wolfowicz, M. , Y. Litmanovitz , D. Weisburd , and B. Hasisi . 2021. “Cognitive and Behavioral Radicalization: A Systematic Review of the Putative Risk and Protective Factors.” Campbell Systematic Reviews 17: e1174. 10.1002/cl2.1174. 37133261 PMC10121227

[cl270041-bib-0029] Yon, Y. , C. Mikton , Z. D. Gassoumis , and K. H. Wilber . 2017. “Research Protocol for Systematic Review and Meta‐Analysis of Elder Abuse Prevalence Studies.” Canadian Journal on Aging/La Revue Canadienne Du Vieillissement 36, no. 2: 256–265. 28399951 10.1017/S0714980817000137

[cl270041-bib-0030] Zych, I. , and E. Nasaescu . 2022. “Is Radicalization a Family Issue? A Systematic Review of Family‐Related Risk and Protective Factors, Consequences, and Interventions Against Radicalization.” Campbell Systematic Reviews 18, no. 3 (July): e1266. 10.1002/cl2.1266. 36913228 PMC9300959

