# Supplementary material for: PROTOCOL: Prevalence and Risk and Protective Factors for Radicalization Among School‐Aged Youth: A Systematic Review
Source: Campbell Syst Rev. 2025 Apr 24;21(2):e70041. doi: 10.1002/cl2.70041 (PMC12018840; doi:10.1002/cl2.70041)
Supplement: Supplementary file 1 — Appendix. Instrument for data extraction of primary studies. [file CL2-21-e70041-s001.docx]

**Appendix. Instrument for data extraction of primary studies**

1) Basic information on each primary study included

- Coder:
- Coding date:

• Study ID (unique ID in database for this primary study):

- Full citation:

• Author(s):

• Publication year:

• Study title:

• Document language (English or other):

• Document type

- Peer reviewed journal article
- Book
- Government report
- Police report
- Technical report
- Conference paper
- Dissertation or thesis
- Other (specify)

• Country where the study was conducted:

• Year(s) in which study was conducted:

- Source of funding (if applicable)
  - - Government
    - Research institution
    - NGO
    - Other

2) Information on the sample for each primary study

• Number of waves/cohorts

- Data for each wave/cohort:

• Sample size

- Total sample size:
- Sample size of comparison group:

• Age

- Age range:
- Mean age:

• Gender

- % Female:
- % Male:
- % Other (specify):

• Racial/Ethnic background

- List all reported groups with percentages available.

• Socioeconomic status

- % High:
- % Medium:
- % Low:
- % Mixed:
- % Other (specify):
- Not reported

• Recruitment strategy

- Describe recruitment approach.
- List settings from where participants were recruited: (e.g., schools, community, youth centers)

• Was attrition a problem?

- Yes (describe)
- No
- N/A
- Initial response rate:

3) Methodology for each primary study

• Study design

- Cross-sectional
- Longitudinal
- Case control
- Other

• If longitudinal, list how many follow-ups and length of follow-up.

• Definition of radicalization used in the study:

• Source of measure used to assess radicalization

- Open source
- Government data
- Self-reported
- Peer/family reported
- Practitioner reported
- Other
- Name of instrument used to assess radicalization, if applicable:
- Ideological strain(s) of radicalization examined
  - - Right-wing
    - Left-wing
    - Incel
    - Religious
    - Ethno-nationalist
    - Other
    - Mixed
    - Unspecified

• Limitations noted by PIs:

4) Risk of bias4a) Prevalence studies (from Rob-PREVmh, Tonia, et al. 2023)

1. *Representation of the prevalence sample*. Was the sample invited to participate in the study a true or close representation of the target population?

- - - High Representation
    - Low Representation
    - Unclear

2. *Representation of the responders*. Was the sample that provided data a true or close representation the sample invited to participate?

- - - High Representation
    - Low Representation
    - Unclear

3. *Measurement of the condition*. Was the condition measured/detected in an unbiased and reproducible way for all participants?

- - - High quality measurement
    - Low quality measurement
    - Unclear

4b) Risk/protective factors (Murray et al 2009 Maryland Quality Scales)

Checklist for risk factors: *design generating risk factor* (3-point scale):

- - 1-crosssectional data
  - 2-retrospective data
  - 3-prospective data

Cambridge Correlate Scale: *Good measure of outcome* (2-point scale)

- - 1=Reliability coefficient > or =.75 and reasonable face validity or criterion or convergent validity coefficient > or =0.3 or more than one instrument or information source used to assess outcome
  - 0=None of the above

Cambridge Correlate scale: *Good measure of correlate* (2-point scale)

- - 1=Reliability coefficient .75 and reasonable face validity or criterion or convergent validity coefficient > or = .30 or more than one instrument or information source used to assess correlate
  - 0=None of the above

Maryland correlates scale: *sampling* (2 point scale)

- - 1=Total population sampling or random sampling
  - 0=Convenience sampling or case–control sampling

Maryland correlates scale*: Adequate sample size scale* (2 point scale)

- - 1=400 and over
  - 0=under 400

Cambridge correlate scale*: Adequate response rates* (2-point scale)

- - 1 Response and retention rates >70% and differential attrition <10%
  - 0 Response rate <70% or retention rate <70% or differential attrition > 10%

IF RELEVANT TO PREVALENCE, GO TO A4. IF RELEVANT TO RISK/PROTECTIVE FACTORS, GO TO B4.

A4) Findings

• Prevalence rate of radicalization (percentage, range):

• Statistical methods used

- Type of analysis conducted (e.g., logistic regression, chi-square):
- Adjustments for confounding variables, if any:

• Key findings reported about prevalence (e.g., observed trends or patterns, associations with specific factors, absence of association, etc.):

• Subgroup analyses (e.g., demographic, geographic, contextual) reported (if any):

B4) Findings

For each risk/protective factor

• How was the risk/protective factor defined?

• How was the risk/protective factor measured (instrument/methods used)?

• What was the source of the risk/protective factor data (e.g., survey, observation) ?

• How was the statistical relationship assessed (e.g., correlation, regression)?

• Adjusted and unadjusted coefficient (value, direction, p-value, analysis sample size):

• Confounders controlled for (if any):

• Subgroup analyses reported (if any):

• What PI(s) states about finding(s):

FOR ALL PRIMARY STUDIES

Reviewer notes:

- Provide additional context or notes relevant to inclusion/exclusion decisions, risk of bias/methods challenges, and potential future research.
